# The potential neuroprotective of luteolin against acetamiprid-induced neurotoxicity in the rat cerebral cortex

**DOI:** 10.3389/fvets.2024.1361792

**Published:** 2024-05-02

**Authors:** Ashraf Albrakati

**Affiliations:** Department of Human Anatomy, College of Medicine, Taif University, Taif, Saudi Arabia

**Keywords:** luteolin, acetamipripd, oxidative stress, neurotoxicity, cerebral cortex

## Abstract

Acetamiprid is a class of neuroactive insecticides widely used to control insect pests. The current study aimed to investigate the potential neuroprotective effects of luteolin against acetamiprid-induced neurotoxicity in the rat cerebral cortex. Four equal groups of adult male rats (10 in each): control, acetamiprid (40 mg/kg for 28 days), luteolin (50 mg/kg for 28 days), and acetamiprid+luteolin cotreatment were used. Acetamiprid was shown to alter the oxidative state by increasing oxidant levels [nitric oxide (NO) and malondialdehyde (MDA)] and decreasing antioxidants [glutathione (GSH), glutathione peroxidase (GPx), glutathione reductase (GR), superoxide dismutase (SOD), and catalase-(CAT)], with increased activity of nuclear factor erythroid 2–related factor 2-(Nrf2). Likewise, acetamiprid increases the inflammatory response, as evidenced by increased interleukin-1β (IL-1β), tumor necrosis factor-α (TNF-α), and nuclear factor kappa B-(NF-κB). In contrast, the treatment with luteolin brought these markers back to levels close to normal, showing that it protects neurocytes from oxidative damage and the neuroinflammation effects of acetamiprid-induced inflammation. Luteolin also demonstrated a neuroprotective role via the modulation of acetylcholinesterase (AChE) activity in the cerebral cortex tissue. Histopathology showed severe neurodegenerative changes, and apoptotic cells were seen in the acetamiprid-induced cerebral cortex layer, which was evident by increased protein expression levels of Bax and caspase-3 and decreased Bcl-2 levels. Histochemistry confirmed the neuronal degeneration, as proven by the change in neurocyte colour from brown to black when stained with a silver stain. Luteolin may have a neuroprotective effect against biochemical and histopathological changes induced by acetamiprid in the rat cerebral cortex.

## 1 Introduction

Neurotoxicity occurs through exposure to several toxic agents, such as pesticides, that change the histoarchitecture and function of the nervous tissue (1). Numerous prior studies have indicated a relationship between the excessive application of pesticides and brain diseases (2, 3). Furthermore, direct or indirect exposure to pesticides causes many toxic symptoms, including respiratory failure, neuromuscular paralysis, and convulsions.

Acetamiprid is a neonicotinoid compound typically used to manage insects and mitigate their effects on vegetables and fruits (4). It induces insect paralysis and death by agonising on nicotinic acetylcholine receptors (5). In this regard, it has been reported that even low concentrations of indirect exposure to acetamide-contaminated food and water can induce various neurogenic diseases (6).

Prolonged exposure to acetamiprid increases oxidative stress, inflammation, and apoptosis, leading to organ damage (7). Moreover, other studies have shown that extended exposure to acetamiprid leads to significant histopathological changes in the brain (8), liver (8), and kidney (9). This occurs by increasing the production of reactive oxygen species (ROS) and disrupting the balance of oxidative processes (10). Acetamiprid is broken down in the liver to make 5-hydroxyimidacloprid. This compound raises ROS levels and stops antioxidant enzymes from releasing lipid peroxide, which increases oxidative stress. Moreover, the elevation of ROS inside the nucleus has detrimental effects on the structure of DNA, leading to abnormal gene expression (10, 11).

Acute activation of inflammation in neural tissue, which is often a protective reaction to different stimuli, may be harmful, particularly when caused by certain chemical exposures. Acetamiprid has been discovered to activate inflammatory pathways in neuronal cells, producing pro-inflammatory cytokines and mediators (12). The initial protective reaction might result in persistent neuroinflammation, contributing to neuronal damage and degeneration (13). In addition, acetamiprid has been implicated in activating apoptosis in nervous tissue. Acetamiprid’s disruption of crucial signaling pathways in neurons may trigger apoptosis, resulting in cellular demise and possibly playing a role in neurodegenerative disorders (12). Like other neonicotinoid compounds, acetamiprid inhibits AChE, accumulating acetylcholinesterase at the cholinergic synapses, inducing neurotoxicity, and eventual neurodegeneration (14).

Luteolin has several pharmacological activities and antioxidant properties associated with scavenging oxygen species (15). Moreover, multiple studies have documented luteolin’s beneficial effects in treating several neural diseases (16, 17). Luteolin protects against lead-induced testicular toxicity by preventing oxidative stress, inflammation, and apoptosis, thus improving degenerative changes in testicular tissue in rats (18). Similarly, luteolin was foun effective against bisphenol-induced renal toxicity due to the activation of anti-oxidation and anti-inflammatory mediators (19). Luteolin is also neuroprotective in mice’s brains from sodium nitroprusside-induced oxidative damage by scavenging and chelating effects (20). In addition, luteolin was found to be effective in treating neurodegenerative disorders in lipopolysaccharide-activated microglia through decreasing proinflammatory and proapoptotic gene expression (21). Furthermore, luteolin has been proven to have anti-inflammatory effects against brain injury induced by fibrillary Aβ1-40 (22). Likewise, luteolin was also found to have a neuroprotective role against ischemic stroke by inhibiting apoptosis and oxidative damage in keratinocytes (23). So, according to these previous studies, the study aimed to evaluate the possible neuroprotective role of luteolin against acetamiprid-induced neurotoxicity and regenerate the neurocytes in the rat cerebral cortex.

## 2 Materials and methods

### 2.1 Experimental animals and study design

Forty male Wistar rats (weight 150–200 g) were purchased from the animal unit of King Abdulaziz University, Saudi Arabia. Rats were acclimatized for 1 week before the start of the study. Rats were placed in cages (12-h light/dark cycle) at room temperature. The rats had free access to standard laboratory food and water *ad libitum* throughout the experiment. The research ethics committee approved all experimental protocols for laboratory animal care, Taif university (approval no. 43–068).

### 2.2 Animal groups

Rats were split into four groups of 10 each:

- Control group: rats were given physiological saline (0.9% NaCl) for 28 days.
- Luteolin group: according to Li et al. (24), rats were given luteolin (50 mg/kg) daily for 28 days. Luteolin administration at a dose of 50 mg/kg showed potent anti-inflammatory and antioxidant activity in colitis model induced in rats (24).
- Acetamiprid group: according to Dhouib et al. (25), rats were orally gavaged with acetamiprid (40 mg/kg) for 28 days. Dhouib et al. (25) showed that acetamiprid administration at a dose of 40 mg/kg induced neurotoxicity through enhancing neuronal oxidative stress, inflammatory response, impaired motor coordination and caused histopathological changes
- Acetamiprid+luteolin group: rats were orally gavaged 40 mg/kg of acetamiprid an hour before 50 mg/kg of luteolin for 28 days.

All treatments lasted for 28 consecutive days. On day 28, rats in all the groups were killed, the brains were collected from the rats, and each rat’s cerebral cortex layer was dissected for histopathology, histochemistry, and immunosorbent assay.

### 2.3 Chemicals

Acetamiprid-N-desmethyl (190604–92-3) and luteolin (CAS Number: 491–70-3) were purchased from Sigma-Aldrich, United States.

### 2.4 Estimation of redox status

#### 2.4.1 Oxidative stress index evaluation

A spectrophotometer (UV–Vis Spectrophotometer from LAB-KITS) was used to measure antioxidant activity in this study. Malondialdehyde (MDA) Cat.No.: E-BC-K025-M was measured as a lipid peroxidation (LPO) using the thiobarbituric acid according to Ohkawa, Ohishi (26). Based on Ellman’s method, the level of decreased GSH Cat Number: E-BC-K096-S in the brain homogenates was evaluated (27). Nitric oxide (NO) Cat Number: E-BC-K035-S level was measured using Griess reagent in accordance with the method described by Green, Wagner (28).

#### 2.4.2 Antioxidant activity assessment

The activities of glutathione peroxidase (Cat Number: E-BC-K096-S) and glutathione reductase (Cat Number: E-BC-K096-S) in the cerebral cortex was estimated using the methods of Paglia and Valentine (29) and De Vega, Fernandez (30), respectively. Cerebral cortex superoxide dismutase (SOD) Cat Number: E-BC-K019-S activity was tested based on the method of Nishikimi et al. (31). Catalase (CAT) Cat Number: E-BC-K031-M activity was estimated according to the procedures described by Aebi (32). All kits were purchased from Elabscience Bionovation, Inc., United States. The levels of Nrf2 (catalog number: MBS752046, MyBioSource, KSA) was determined as described by the manufacturer’s kit. Cerebral cortex protein levels were determined for all measurements using the protocols described by Bradford (33).

### 2.5 Estimation of inflammatory cytokines

ELISA was used to measure levels of inflammatory cytokines in this study. Tumor necrosis factor-α (TNF-α) (catalog number: CSB-E11987r, Cusabio Biotech Co., Ltd.), interleukin-1β (IL-1β) (catalog number: E-EL-R0012, Elabscience Biotechnology Co., Ltd., Wuhan, China), and nuclear factor kappa B (NF-κB) (catalog number: E-EL-R0674, Elabscience Biotechnology Co., Ltd., Wuhan, China) were assayed using ELISA kits supplied by the manufacturer’s instructions.

### 2.6 Assessment of proapoptotic and antiapoptotic proteins

Cerebral cortex apoptotic proteins were assessed using ELISA kits for Bax and Bcl-2 (BioVision, Inc., Milpitas, CA, United States; catalog numbers E4513 and CSB-E08854r, respectively). A colourimetric kit was used to measure caspase-3 activity (Sigma-Aldrich: CASP3C-1KT). All assays were performed according to the manufacturer’s instructions.

### 2.7 Determination of acetylcholinesterase (AChE) in the cerebral cortex tissue

Acetylcholinesterase (Cat Number: E-BC-K174-M, Elabscience Bionovation, Inc., United States) activity in the cerebral cortex was measured using the method explained by Ellman, Courtney (34). Based on the yellow colour that appeared after the addition of thionitrobenzoic acid, measured at 412 nm, acetylcholinesterase activity was identified.

### 2.8 Histopathological and histochemical examinations of the cerebral cortex

#### 2.8.1 Histopathology examination

For histopathological examination of the cerebral cortex, brains were immediately removed after killing the rats, and the cerebral cortex was carefully separated from the brain. The samples of the cerebral cortex were fixed in 10% neutral formaldehyde for 24 h and dehydrated using high-grade alcohol within paraffin, and then the cerebral cortex was cut by microtome (Leica) into 5-μm thickness. Specimens were stained with hematoxylin and eosin (35). Finally, the slides were examined using a Nikon Eclipse E200-LED (Tokyo, Japan) microscope.

#### 2.8.2 Histochemistry examination

This study examined neuronal degeneration in the cerebral cortex tissue using silver staining as a specific stain for the degenerative tissue, especially in nervious tissue (36). A small part of the cerebral cortex tissue was deparaffinized, rehydrated, and incubated in 20% silver nitrate in the dark for half an hour at 37°C. Then, the slides were added to a solution of ammonium hydroxide + silver nitrate for 15 min in the dark. The developer (20 mL 37% unbuffered formalin, 100 μL nitric acid+0.5 mg citric acid in 100 mL of distilled water) was added to the silver nitrate solution and used to stain the slides before fixation with 5% aqueous sodium thiosulfate (37).

### 2.9 Statistical analyses

All results were expressed as the means ± SD. Data for multiple variable comparisons were analyzed by one-way analysis of variance (ANOVA), followed by Tukey HSD post-hoc test, using the statistical package SPSS. The acceptable level of significance was set at *p* < 0.05.

## 3 Results

### 3.1 Effect of the luteolin treatment against the oxidant/antioxidant status following acetamiprid exposure of the cerebral cortex

**Figure 1:**
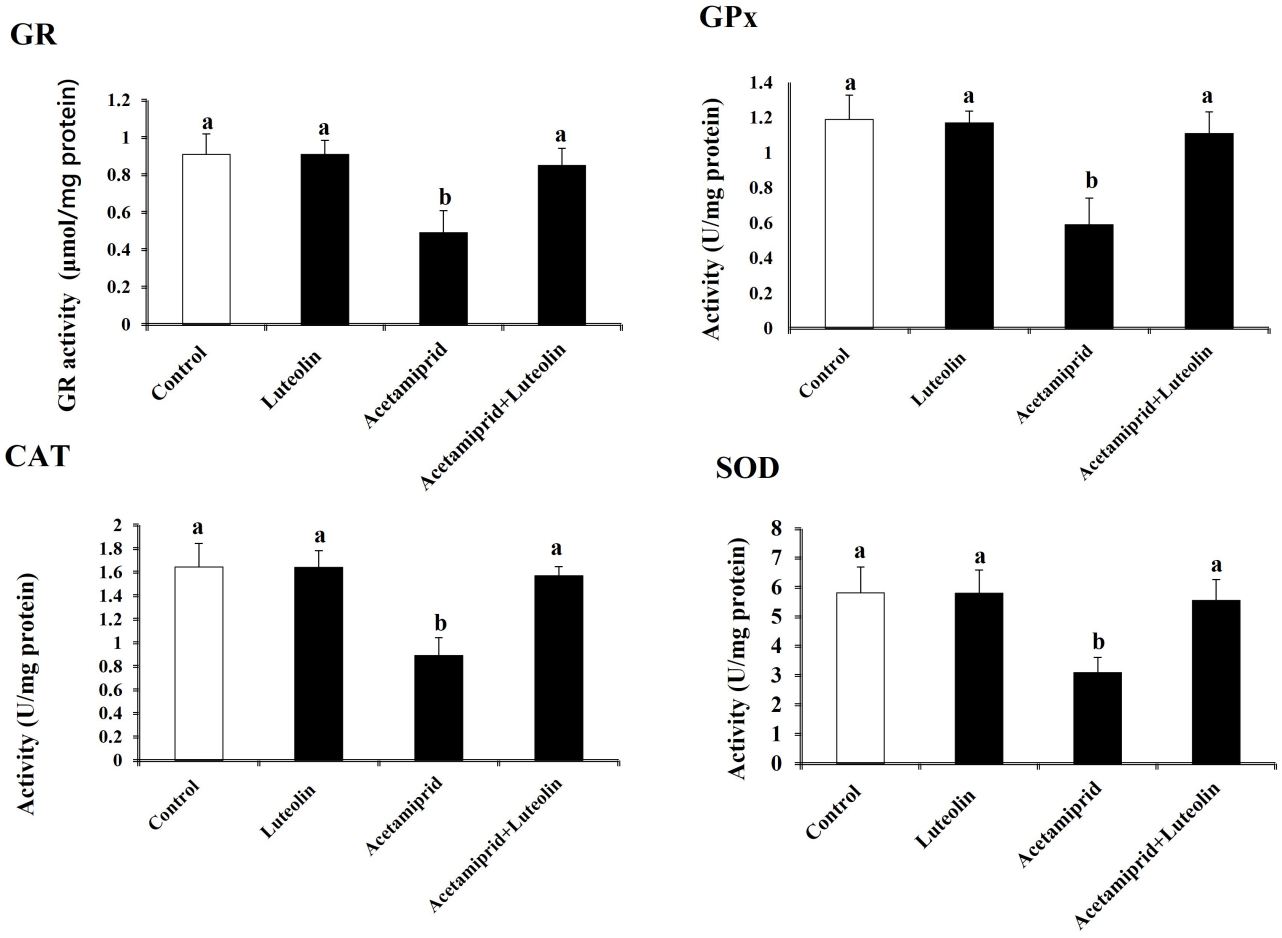
Effects of orally administered luteolin on antioxidant enzymes, GPx, GR, SOD, and CAT in cerebral cortex tissue following acetamiprid exposure. Results are shown as the mean ± standard deviation (SD). a and b indicate significant differences (*p* < 0.05) compared with the control and acetamiprid-treated groups.

**Figure 2:**
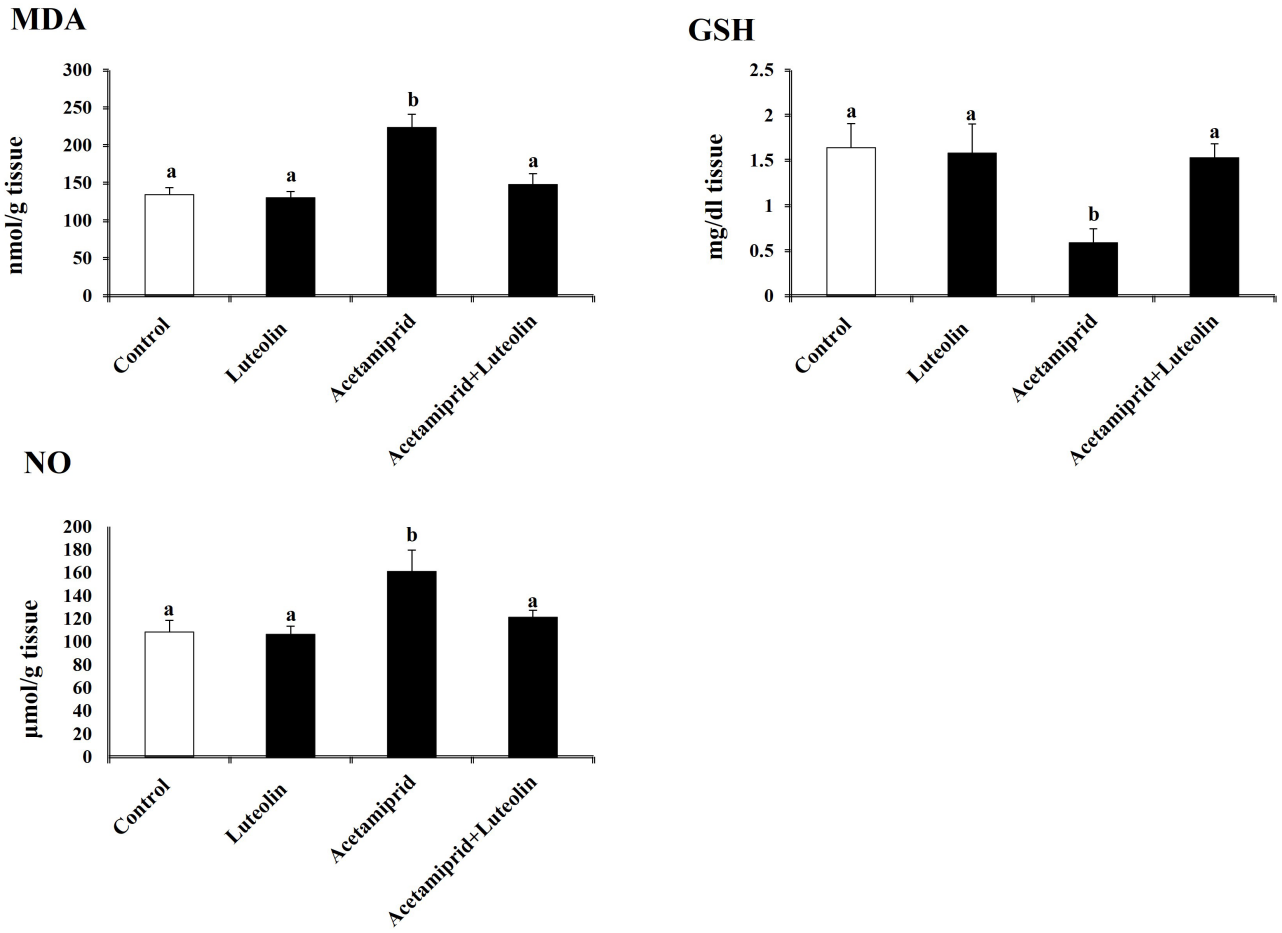
Effects of orally administered luteolin on NO, MDA, and GSH in cerebral cortex tissue following acetamiprid exposure. Results are shown as the mean ± standard deviation (SD). a and b indicate significant differences (*p* < 0.05) compared with the control and acetamiprid-treated groups.

Levels of pro-oxidant and antioxidant molecules were measured to evaluate the effect of luteolin treatment on the oxidative state in the cerebral cortex following acetamiprid administration. The activities of cerebral cortex redox antioxidant enzymes, as seen in Figure 1, shows that the antioxidant enzymes SOD, CAT, GPx, and GR were significantly less active (*p* < 0.05) in the group that was given acetamiprid compared to the group receiving no treatment. In contrast, rats pre-treated with luteolin exhibited a noteworthy increase in the enzymatic activity of all the enzymes examined compared to the group that received treatment alone. On the other hand, levels of oxidative biomarkers in rat cerebral cortex tissue, as seen in Figure 2, demonstrated a statistically significant elevation (*p* < 0.05) in the level of MDA and NO, while concurrently seeing a notable reduction in the levels of GSH in the rats treated with acetamiprid in comparison to the control rats. However, luteolin raised GSH levels and decreased nitric oxide and MDA levels in comparison to the treated group. Nevertheless, the administration of luteolin effectively mitigated the acetamiprid-induced oxidative stress in the cerebral cortex tissue of rats by augmenting the antioxidant defence mechanism against harmful free radicals.

**Figure 3:**
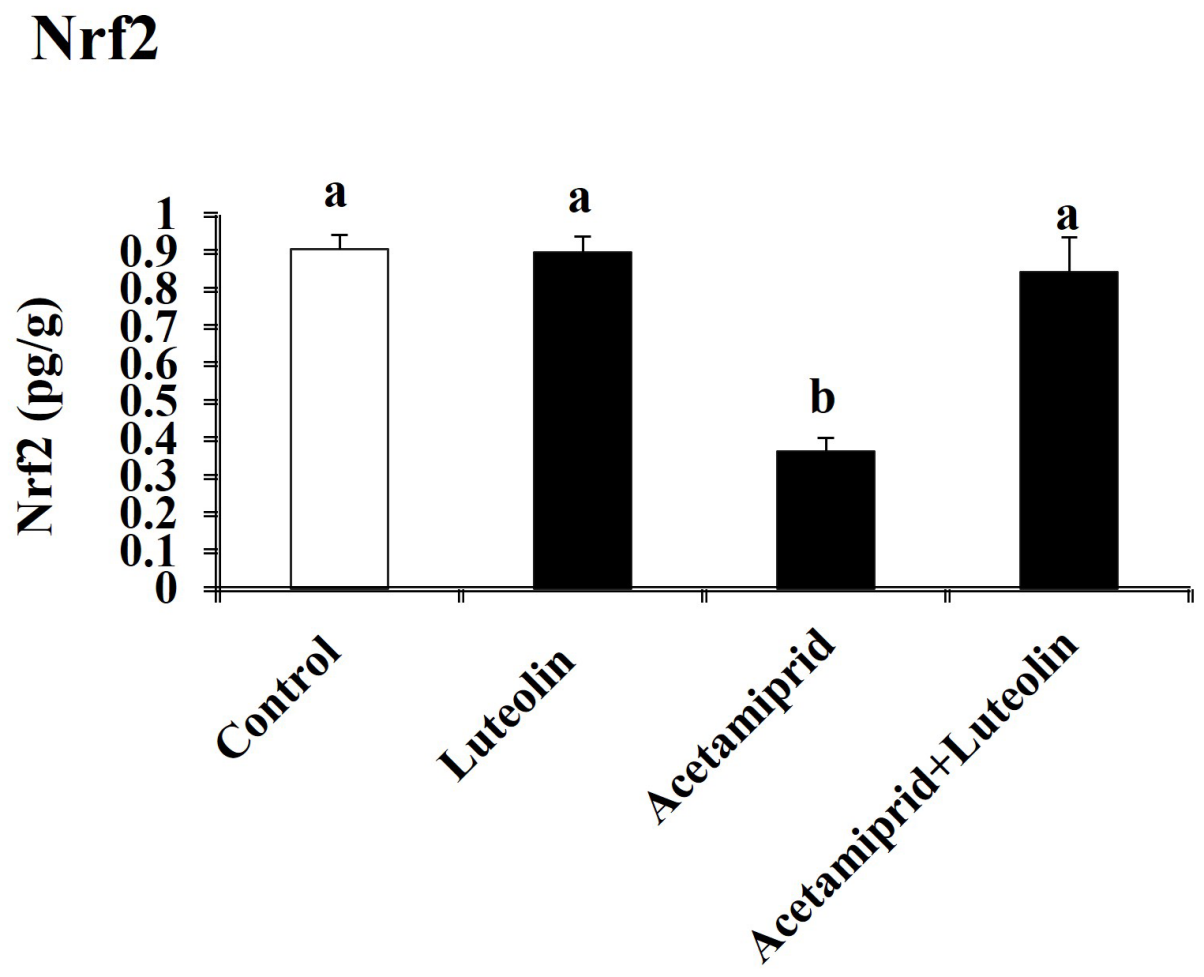
Effects of orally administered luteolin on Nrf2 in cerebral cortex tissue following acetamiprid exposure. Results are shown as the mean ± standard deviation (SD). a and b indicate significant differences (*p* < 0.05) compared with the control and acetamiprid-treated groups.

To determine the molecular antioxidant mechanism, the Nrf2 level in cerebral cortex tissue was quantified. The ELISA results indicated that the concentrations of Nrf2 decreased significantly (*p* < 0.05) after exposure to acetamiprid compared to the control group. No statistically significant alteration was observed in the activity of these antioxidant molecules in the group that received luteolin treatment. In contrast, the administration of luteolin prior to exposure to acetamiprid resulted in a substantial increase (*p* < 0.05) in the concentrations of Nrf2-inactivated SOD, CAT, and GPx in the cerebral cortex tissue, as compared to their activities in the group exposed to acetamiprid (Figure 3).

### 3.2 Effect of the luteolin administration on the neuroinflammatory status of the cerebral cortex following acetamiprid administration

**Figure 4:**
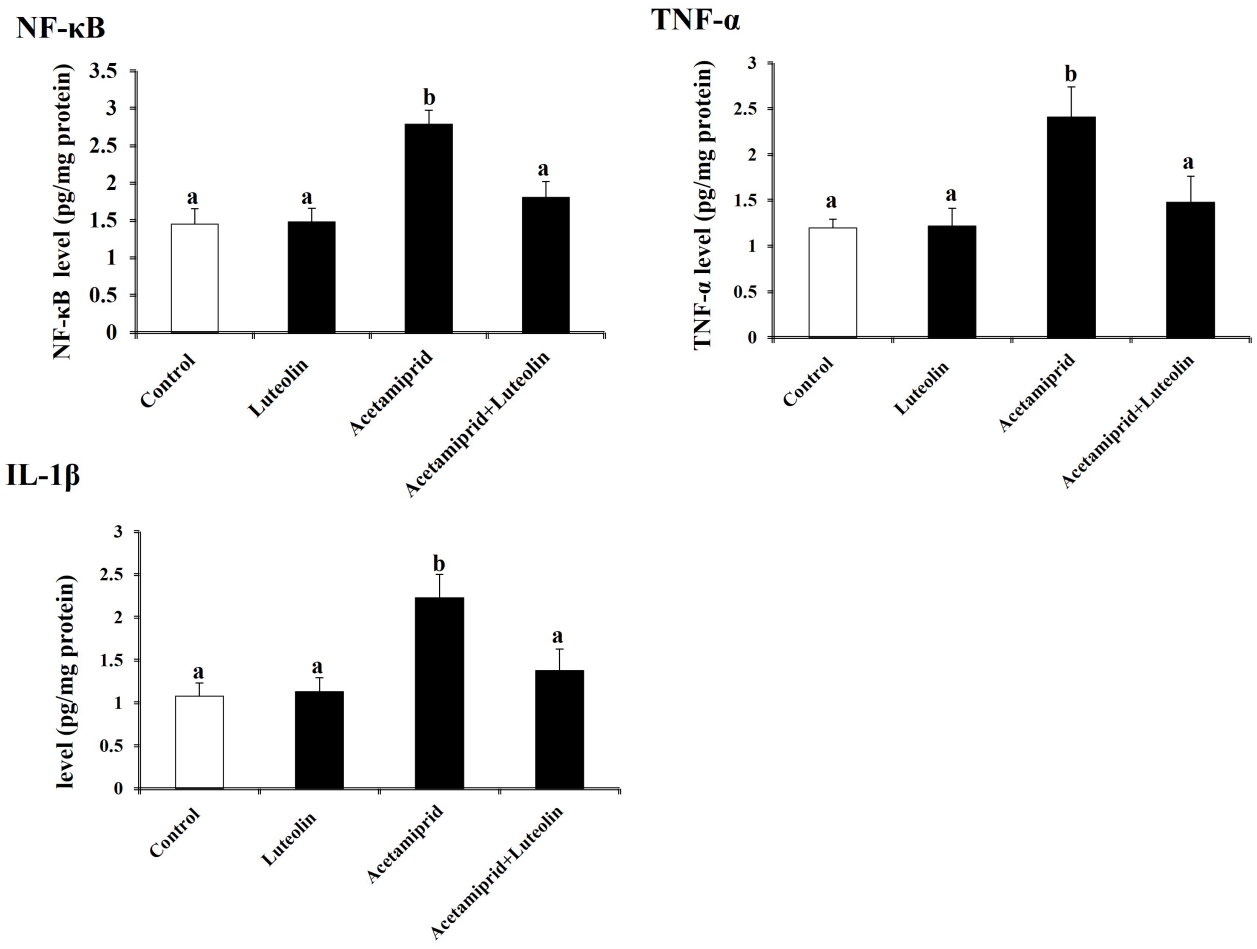
Effects of orally administered luteolin on the levels of inflammatory mediators (TNF-α, IL-1β, and NF-κB) in cerebral cortex tissue following acetamiprid exposure. Results are shown as the mean ± standard deviation (SD). a and b indicate significant differences (*p* < 0.05) compared with the control and acetamiprid-treated groups.

Neuroinflammation biomarkers showed that acetamiprid-treated rats displayed notably (*p* < 0.05) increased levels of proinflammatory cytokines (TNF-α, IL-1β and NF-κB) in cerebral cortex tissues as compared with control rats. Contrariwise, a significant decrease (*p* < 0.05) of TNF-α, IL-1β and NF-κB was shown in the luteolin-treated rats after acetamiprid treatment compared to the acetamiprid group (Figure 4).

### 3.3 Effects of luteolin treatment on apoptotic proteins following acetamiprid exposure

To examine neuronal apoptotic effects in the acetamiprid-treated rats and the possible antiapoptotic role of luteolin treatment. The levels of Bcl-2, Bax, and caspase-3 activity were investigated in the cerebral cortex of rats in all groups. The apoptotic protein results demonstrated a significant elevation (*p* < 0.05) in the levels of apoptotic proteins (Bax and caspase-3) in acetamiprid-treated rats compared with control rats. In contrast, a significant decrease (*p* < 0.05) in the Bcl-2 level (antiapoptotic protein) was shown in acetamiprid-treated rats compared with control rats.

**Figure 5:**
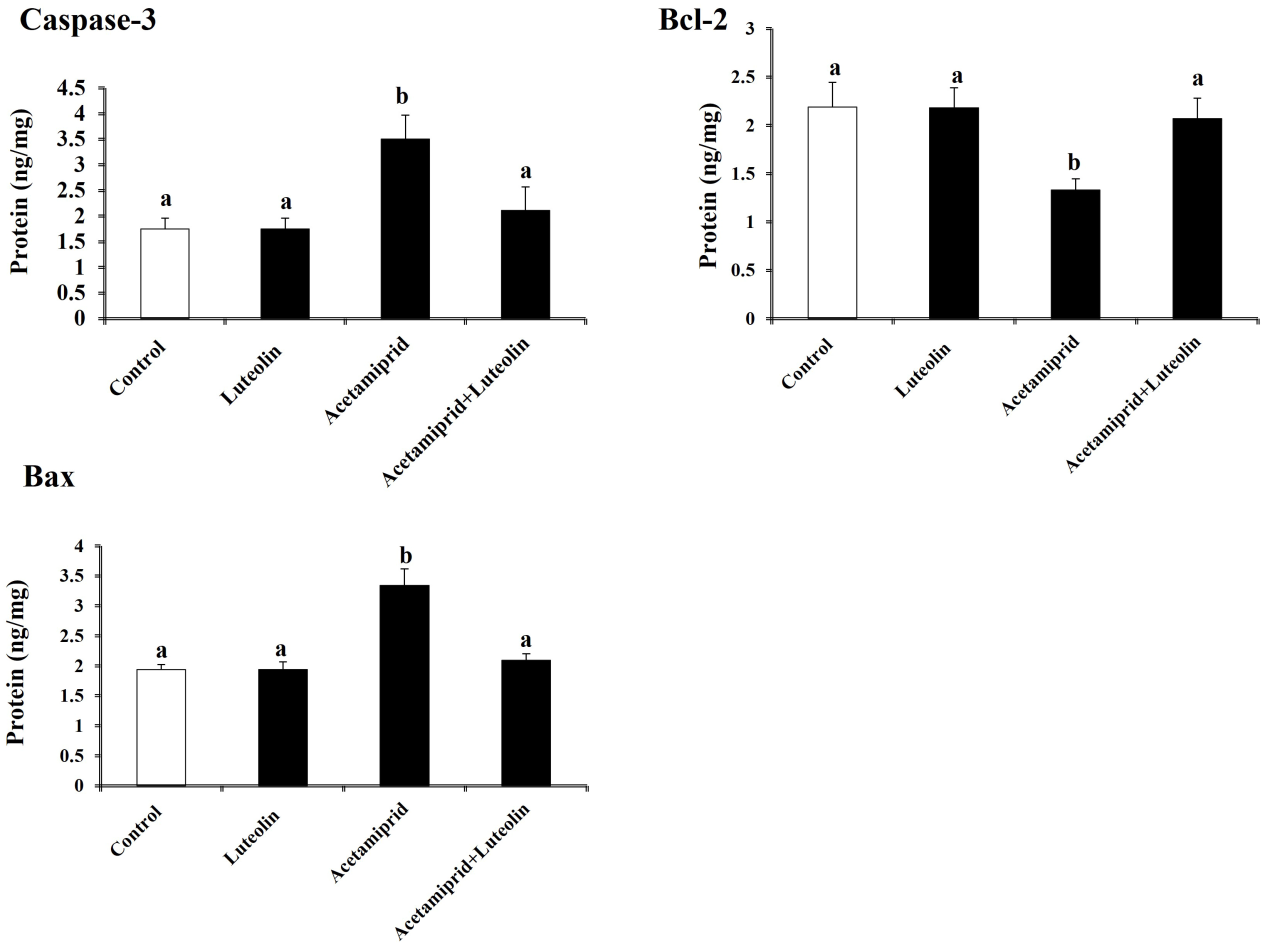
Effects of orally administered luteolin on the levels of apoptosis markers, caspase-3, Bax and Bcl-2 in cerebral cortex tissue following acetamiprid exposure. Results are shown as the mean ± standard deviation (SD). a and b indicate significant differences (*p* < 0.05) compared with the control and acetamiprid-treated groups.

Nevertheless, the administration of luteolin effectively inhibited the apoptotic cascade, restored the abnormal levels of apoptotic proteins to their baseline values, and counteracted the changes in apoptotic proteins induced by exposure to acetamiprid, as evidenced by a comparison with untreated acetamiprid levels. These findings suggest that luteolin plays a protective role in preventing neuronal loss following exposure to acetamiprid, as depicted in Figure 5.

### 3.4 Effects of luteolin treatment on AChE activity following acetamiprid exposure

**Figure 6:**
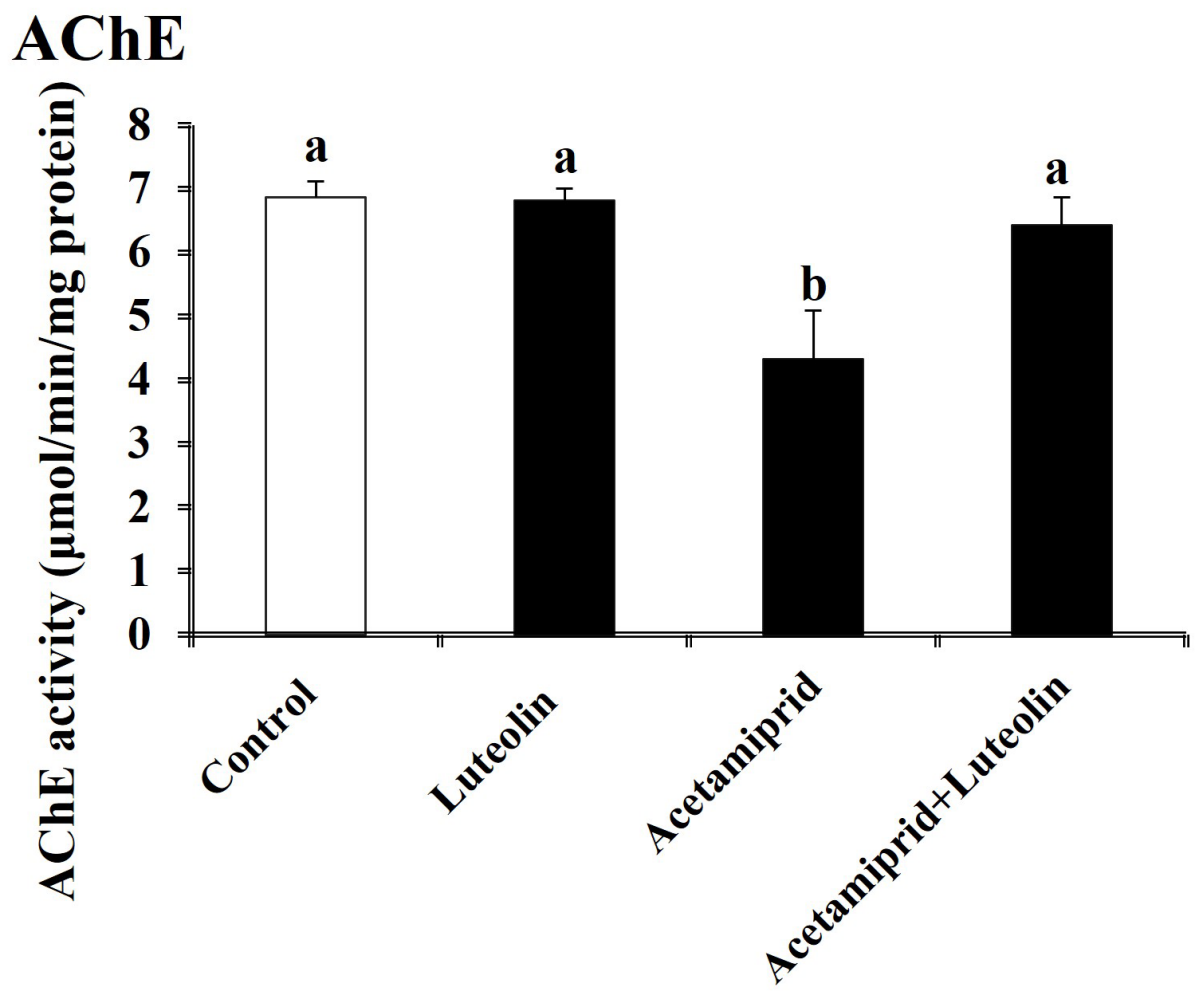
Effects of orally administered luteolin on AChE activity in cerebral cortex tissue following acetamiprid exposure. Results are shown as the mean ± standard deviation (SD). a and b indicate significant differences (*p* < 0.05) compared with the control and acetamiprid-treated groups.

The AChE activity was investigated in the cerebral cortex tissue of rats in all groups. The acetamiprid-treated rats presented a significant (*p* < 0.05) increase in AChE activity in comparison with rats in the control group. This result indicates that acetamiprid exposure led to reduced levels of acetylcholine in cerebral cortex tissue. However, luteolin treatment restored AChE activity to near-normal levels, indicating the potent neuromodulator role played by luteolin against neurotransmitter damages in cerebral cortex tissue induced-acetamiprid. A significant decrease in AChE activity was seen in the rats treated with luteolin compared to those in the acetamiprid group (Figure 6).

### 3.5 Effects of luteolin treatment on neuropathological alterations in the cerebral cortex neurocytes following acetamiprid exposure

**Figure 7:**
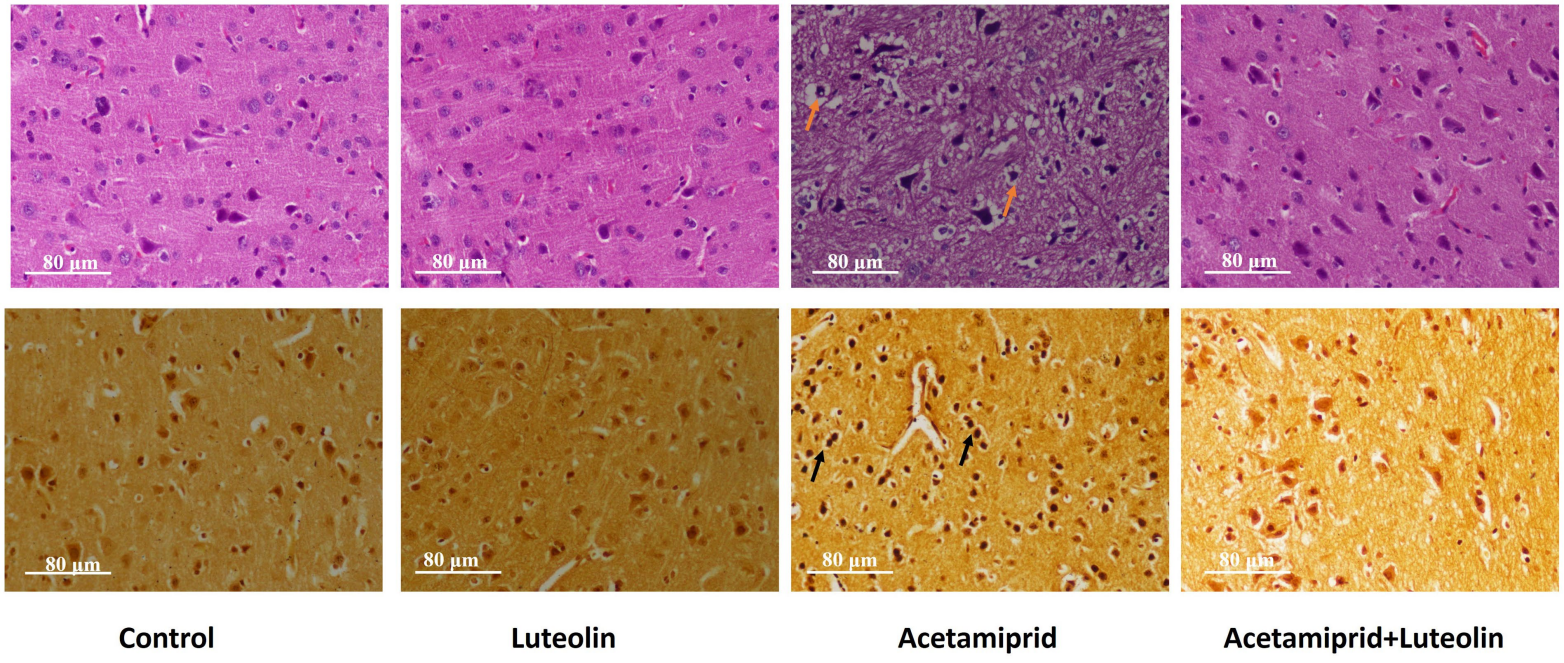
Effects of orally administered luteolin on the histopathological changes top panel for cerebral cortex by using hematoxylin and eosin stains, and bottom panel by using silver stain in cerebral cortex tissue following acetamiprid exposure.

Histological examination of rats in the control and luteolin groups showed the typical architecture of the cerebral cortex neurocytes. Cortical histopathological changes following acetamiprid exposure revealed severe atrophied, shrunken neurocytes with degenerative vacuolar changes and hyperchromatic nuclei. Additionally, apoptotic cells were also seen. Also, some neurocytes appeared to have vacuolation of the neuropil (Figure 7). Conversely, luteolin treatment in rats pretreated with acetamiprid restored histopathological alterations in rats in the cerebral cortex, evidenced by the few necrosis, vacuolation in the neuropil, and remarkable regression of the degenerative changes and pyramidal neurons accompanied by decreased perineuronal haloes induced by acetamiprid (Figure 7).

To confirm that neuronal degeneration was observed in the cerebral cortex tissue by histological examination following acetamiprid exposure, the silver stain was used as a specific marker to identify neuronal degeneration in the cerebral cortex tissue following acetamiprid exposure (38).

Histochemical examination of the cerebral cortex confirms neural degenerative changes in the cerebral cortex tissue following acetamiprid exposure; neurocytes appeared argyrophilic (neurocyte contents stained with darker brown or black by silver salts). On the other hand, luteolin treatment in rats pretreated with acetamiprid restored neural degenerative alterations in the cerebral cortex, as evidenced by the change in the colour of the neurocytes from black to light brown (Figure 7).

## 4 Discussion

The cerebral cortex layer is the most significant part of the brain, which contributes to higher-order brain functions, including learning, decision-making, emotion, and language (39). The cerebral cortex is histologically classified into six layers based on neurocyte types. Therefore, any alteration in the histological structure of the cerebral cortex cells induced by the toxic agents leads to the loss of many neuronal tasks like learning and spatial memory (40).

Acetamiprid exposure, common in agricultural settings, causes oxidative stress, DNA damage, and apoptosis. Therefore, it leads to neurodegenerative diseases (41). The present study evaluated the redox state, revealing elevated MDA and NO levels combined with a significant reduction in GSH, GPx, GR, and SOD in the cortical tissue following acetamiprid administration. In this regard, several studies have indicated that acetamiprid has toxic effects on the brain and that it induces oxidative stress due to increased production of ROS and LPO (42, 43). Oxidative stress plays a significant role in the process of tissue damage. It occurs when an imbalance between the production of reactive oxygen species (ROS) and the body’s ability to neutralize or repair their harmful effects arises (44–47). The overproduction of oxidative stress leads to several brain diseases (48, 49). Similar results were observed by Gasmi, Rouabhi (50), who reported that administration of acetamiprid leads to a decrease in the reduced glutathione and activity of glutathione peroxidase and catalase in the brains of rats. Also, they noted a rise in the enzyme activity of glutathione s-transferase and the rate of MDA.

The structural and functional abnormalities in nerve tissue after exposure to neonicotinoid insecticides are attributed to the depletion and inactivation of thiol-containing proteins (51). Glutathione and its derived enzymes are the first lines of antioxidant defence against toxic agents-induced oxidative stress. The depletion of glutathione enzymes may occur due to their conjugation with acetamiprid metabolites, which hinders the generation of ROS and, therefore, impedes oxidative damage (52). Further, it has been suggested that the overproduction of ROS inactivates SOD and CAT via downregulating their protein and mRNA expression upon exposure to neonicotinoids (52). The depletion of antioxidant proteins in neural tissue occurs via the inactivation of Nrf2, a transcription factor that controls the expression of genes associated with antioxidants (53).

MDA is a lipoperoxidation by-product that is powerfully used as a marker of the extreme production of peroxide radicals (54). Acetamiprid promotes the oxidative response by increasing the generation of reactive oxygen species, natural antioxidants necessary for free radical scavenging (6). Neonicotinoid insecticide acetamiprid targets neuronal signal transmission-critical nicotinic acetylcholine receptors (nAChRs) (55). Acetamiprid’s activation of these receptors in the nervous system may affect nitric oxide production (56). Neurovascular regulation, neurotransmission, and neuroplasticity depend on nitric oxide, a versatile nervous system signaling chemical (57, 58). Neonicotinoid insecticide interactions with nAChRs modify NO levels by affecting neuronal nitric oxide synthase (nNOS), which produces NO in neurons (59).

Luteolin is a flavone compound that exists naturally in many plant species, such as carrots and olive oil, and contains a range of pharmacological attributes, notably neuroprotective effects (15). In the presented study, luteolin administration effectively mitigates the oxidative stress induced by acetamiprid, a feature underscored by the significant enhancement in the activities of antioxidant enzymes such as SOD and CAT. This rise in antioxidant enzyme activity shows that luteolin may be able to improve the brain’s natural antioxidant defence system, lowering the harmful effects of free radicals and oxidative stress. Further delving into the molecular aspects of this protection, the current study observed a notable increase in Nrf2 levels following luteolin treatment. The Nrf2 pathway is essential for protecting cells from oxidative stress (60), and luteolin stimulation indicates a specific method by which luteolin increases the body’s endogenous antioxidant defences (61). Nrf2 is recognized for its ability to provide cellular protection against ROS through many mechanisms (62). These mechanisms include enhancing the synthesis of glutathione, upregulating enzymes involved in antioxidant defence and detoxification, and facilitating the degradation of superoxide and peroxide radicals by glutathione peroxidase and superoxide dismutase.

Several studies have reported that exposure to neonicotinoid insecticides increases ROS, activating inflammatory pathways in neuronal cells and producing pro-inflammatory cytokines (63). The present study showed a significant increase in proinflammatory mediators, including TNF-α, NF-κB and IL-1β in acetamiprid-exposed rats. Our results indicated a marked decrease in proinflammatory cytokines in lutein-treated rats following acetamiprid exposure compared to acetamiprid-exposed rats. Luteolin contains bioactive elements that neutralize free radicals by lowering lipoperoxidation and increasing antioxidant enzyme activity (64). Lutein may protect against neurotoxicity by neutralizing oxidative stress and reducing acetamiprid-induced neuroinflammatory reactions. Luteolin reduces inflammation by decreasing TNF-α, IL-1, and cyclooxygenase-2 activity and deactivates iNOS in brain tissue after acetamiprid treatment (65). The present study suggests that lutein reduces oxidative stress by reducing MDA and NO. The early defensive response of inflammation might eventually lead to the development of acute neuroinflammation, contributing to the damage and deterioration of neurons (66). In this regard, oxidative stress and possible changes in the homeostasis of cells could cause NF-κB pathways to be activated in nerve tissue (67).

Acetamiprid exposure increases proapoptotic proteins like Bax and caspase-3 and decreases the antiapoptotic protein Bcl-2 (12), which leads to oxidative stress and inflammatory pathways.

Several prior studies have indicated that neurodegenerative diseases resulting from neonicotinoid exposure correlate with increased ROS production levels and activation of TNF-α, caused by reduced levels of Bcl2 and the increase of Bax and caspase-3 (12, 59). Luteolin treatment effectively reversed these changes, highlighting its role in inhibiting the apoptotic pathways activated by acetamiprid, as revealed by the reduced expression proteins of Bax and caspase-3, along with the increased expression proteins of Bcl-2. This suggests that luteolin not only prevents cell death but also promotes cell survival in the context of pesticide-induced neurotoxicity.

Neonicotinoid insecticides are implicated in impacting neuronal nicotinic acetylcholine receptors in nervous tissue (59). Recent studies have indicated that neonicotinoid treatment decreased cholinergic neurotransmitters in the nervous tissue by inhibiting the enzyme AChE (41, 55). Acetamiprid exposure decreased acetylcholine activity, which could disrupt normal neurotransmitter function (68). In this context, it has been reported that an increase in acetylcholine activity is linked with several brain diseases, such as epilepsy disease (69).

In contrast, this study showed that luteolin’s to restore acetylcholine activity to near-normal levels underlines its neuromodulatory role, further protecting against neurotransmitter imbalances caused by acetamiprid. Yu et al. (70) also found that pre-treatment with luteolin had a big effect on the levels of monoamines and activated AChE to protect rats from amyloid-β peptide-induced cognitive impairment. These results agree with the results of this study.

In the current study, histopathology showed that acetamiprid administration for 28 days induced degenerative changes in neurocytes along with apoptotic cells in the pyramidal layer of the cerebral cortex. Furthermore, the pyramidal cells showed irregularly stained nuclei, and other neurocytes appeared to have vacuolated foci with cellular loss compared to the non-treated rats. The findings of this study are consistent with the present study by Khovarnagh and Seyedalipour (8), who reported that acetamiprid altered the histo-architecture of the rat brain and induced damage including gliosis, hyperemia, and necrosis. In contrast, histopathology examination in luteolin showed enhancement of the histo-structural structure of the cerebral cortex, as evidenced by the few necrotic cells and remarkable improvement of the degenerative changes and pyramidal neurons, accompanied by decreased apoptotic cells.

The current study used the silver stain to confirm the cortical neurodegenerative changes. Histochemical examination confirmed neural degenerative changes following acetamiprid exposure; neurocytes appeared darker brown or black. In addition, consistent with expectations, the histochemistry examination showed the transformation of neurocytes from a black stain to a light brown shade, indicating an amelioration of neurocyte degeneration within the cortical layer.

## 5 Conclusion

The study determined that luteolin has neuroprotective properties that can counteract the damage caused by acetamiprid in the cerebral cortex. The findings suggest that luteolin acts through multiple pathways, including enhancing antioxidant defences, reducing inflammatory responses, inhibiting apoptotic pathways, normalizing acetylcholine activity, and mitigating histopathological alterations. These multifaceted protective mechanisms position luteolin as a promising candidate for addressing pesticide-induced neurotoxicity, with potential implications for neuroprotective strategies in environmental and occupational health contexts.

## Data availability statement

The original contributions presented in the study are included in the article/supplementary material, further inquiries can be directed to the corresponding author.

## Ethics statement

This study and all experimental procedures were performed according to the principles of the Ethics Committee of Taif University, Taif, Saudi Arabia (Approval No. HAO-02-T-105) which are in line with the Declaration of Helsinki. The study was conducted in accordance with the local legislation and institutional requirements.

## Author contributions

AA: Conceptualization, Data curation, Formal analysis, Funding acquisition, Investigation, Methodology, Project administration, Resources, Software, Supervision, Validation, Visualization, Writing – original draft, Writing – review & editing.
